# Syringing the Ear

**Published:** 1881-07

**Authors:** 


Syringing the Ear.—Let it be laid down as a rule from which there is to be no departure, that no ear should be actively syringed unless there is something in it which it is important to remove. Cold water should never be put into the ear.

